# Mass spectrometry-based discovery and diagnostic validation of *T. cruzi* antigens in the urine of congenitally infected Chagas Disease patients

**DOI:** 10.1371/journal.pntd.0013082

**Published:** 2025-06-16

**Authors:** Kathryn Cassels, Raghad Almofeez, Jessica Roman, Hannah Steinberg, Ahana Byne, Amanda Haymond, Freddy Tinajeros, María Del Carmen Menduiña, Edith Málaga Machaca, Manuela Verástegui, José Luis Ramírez, Lance Liotta, Robert H. Gilman, Alessandra Luchini

**Affiliations:** 1 Center for Applied Proteomics and Molecular Medicine, George Mason University, Manassas, Virginia, United States of America; 2 Department of Microbiology and Immunology, University of Illinois Chicago, Chicago, Illinois, United States of America; 3 Asociación Benéfica PRISMA, Lima, Peru; 4 Hospital Percy Boland Rodríguez, Ministerio de Salud Bolivia, Santa Cruz, Bolivia; 5 Infectious Diseases Research Laboratory, Department of Cellular and Molecular Sciences, Universidad Peruana Cayetano Heredia, Lima, Peru; 6 Biotechnology Center, Instituto de Estudios Avanzados, Caracas, Venezuela; 7 Department of International Health, Bloomberg School of Public Health, Johns Hopkins University, Baltimore, Maryland, United States of America; University of Florida College of Medicine, UNITED STATES OF AMERICA

## Abstract

**Background:**

Caused by the parasite *Trypanosoma cruzi*, Chagas disease affects an estimated 7 million people globally. Diagnosis of Chagas disease in infants is urgently needed, as early detection allows for more effective treatment and reduced mortality. However, current diagnostics are inappropriate for effective detection in infants due to differences in the mechanism of disease in infants and the infant immune system, as well as lack of diagnostic sensitivity and loss to follow up. Studying peripheral biomarkers in urine can leverage physiological concentration in the bladder to increase yield of proteins secreted by pathogen, infected cells, or antigen processed by immune cells residing in different body sites.

**Principal findings:**

We analyzed the urine of a cohort of infants who were congenitally infected with Chagas disease, using a method including affinity enrichment, mass spectrometry, and bioinformatics analysis to characterize the *T. cruzi* secreted peptidome. We identified 198 peptides specific for *T. cruzi* and analyzed them in light of their potential for diagnostic utility. Our protocol revealed that peptides of the hyper-mutating mucin-associated surface protein and trans-sialidase protein families could be identified in patient urine and can serve as diagnostic markers of disease. We developed antibodies against conserved regions of each protein and validated that these antibodies could be used to differentiate the urine of Chagas disease patients (N = 16 cases) from healthy controls (N = 19). By utilizing affinity enrichment sample preprocessing and anti-trans-sialidase and anti-MASP antibodies in tandem, we differentiated cases from controls with 87.5% sensitivity and 94.7% specificity.

**Conclusions/Significance:**

Our work suggests that it is possible to detect *Trypanosoma cruzi* infection directly from a noninvasively collected fluid such as urine. A direct test in urine with this success rate would be well suited for rapid diagnosis in low-resource areas. Further studies to validate this approach are warranted.

## Author summary

There is an urgent need for a sensitive, specific, and rapid test for congenital Chagas disease (CD) infections. A urinary test detecting protein fragments deriving from the causative agent *Trypanosoma cruzi* would be well-suited to congenital CD diagnostics due to the ease of collecting urine from infants and the potential for highly accurate detection, with minimal false positive and false negative results. Here, we present a pipeline that led to the discovery of many *T. cruzi*-specific peptides in urine samples collected from infants born to infected mothers in rural Bolivia. We designed antibodies against the most promising biomarkers and validated those antibodies in patient samples. Due to our use of innovative affinity concentration techniques, which allow for the concentration of low-abundance biomarkers and the exclusion of potentially interfering host proteins, we were able to achieve greater than 85% sensitivity and specificity using these biomarkers. One of these biomarkers has never been used in CD diagnostic testing. We also include experimental evidence validating the reagents for a lateral flow immunoassay, which requires minimal training and equipment to perform and therefore has transformative potential to increase access to care for individuals in rural and remote communities, where CD is most common.

## 1. Introduction

*Trypanosoma cruzi* can be transmitted to humans through the arthropod vectors, organ transplantation, blood transfusion, and maternal-fetal transmission [1]. Efforts to control Chagas disease (CD) have yielded significant success, as evidenced by an 11.3% global decline in estimated prevalence from 1990 to 2019, with cases decreasing from 7,292,889 in 1990–6,469,283 in 2019 [2–4]. The World Health Organization has identified the detection of congenital *T. cruzi* infections in newborns as a critical component of CD control and elimination programs [2,5]. Congenital transmission accounts for 25% of new infections with an estimate of 15,000 infected infants per year in Latin America [6,7]. The earlier congenital infection is detected, the greater the effectiveness and tolerability of microbicidal treatment [8,9]. Current CD diagnostic guidelines recommend two parasitological tests at birth and one month, followed by serology at 8–12 months [10]. A commonly used microscopy-based technique, known as the micromethod, concentrates the peripheral blood via centrifugation and examines the buffy coat [10]. However, microscopy has low sensitivity (<50% in a single specimen), and an algorithm requiring up to three tests over an extended period is impractical, with high risk of loss to follow up (an estimated 80% of infants fail to complete follow up) [10]. Diagnosis is particularly challenging in rural areas where tests are either difficult to access or may be performed with low accuracy [11]. Molecular methods are more sensitive than microscopy; however, their technical demands and high cost prevent routine use in resource-limited settings [10,12–14]. To enable effective CD screening, a test that is sensitive, specific, and practical for field use is needed [15,16].

Urine antigen detection is a promising alternative for improving the diagnosis of congenital *T. cruzi* infection [17,18]. Its non-invasive nature enhances parental acceptance. We and others have performed pre-clinical and clinical studies to evaluate the feasibility to use urine as a diagnostic specimen. Diagnostic sensitivity ranged from 32.6% to 96%, varying based on the infection stage and detection method [17–22]. However, challenges remain in using urine for diagnosis, including limitations in sensitivity and the lack of identification of the optimal antigen [17,18]. Urinary antigens are present in very low concentrations and degrade rapidly after collection. To address this, affinity capture has been proposed for antigen concentration and preservation prior to analytical measurement [17,23,24]. High-affinity chemical probes (KD < 10^−^¹^2^ M), incorporated in a bioseparation material, enable rapid and efficient antigen capture. The captured antigens can then be eluted in a small volume, achieving a significant concentration factor. Identifying a suitable antigen for CD diagnostics requires overcoming obstacles related to parasite lifecycle complexity, antigenic variability, geographical diversity, and host immune response [25,26].

In this study, we utilized human samples from congenitally infected CD infants, affinity concentration techniques, mass spectrometry proteomics, and bioinformatics analysis to identify and verify conserved *T. cruzi* peptides as potential CD biomarkers. These peptides were then used to generate monoclonal antibodies, which were incorporated into an antigen-down immunoassay and a lateral flow immunoassay. The feasibility of these immunoassays was demonstrated in human samples.

## 2. Materials and methods

### 2.1. Ethics statement

Samples were collected under an IRB-approved protocol as approved by the internal review boards at George Mason University (IRBnet number 523059-1) and Johns Hopkins University (IRB00002644). Written informed consent was obtained from pregnant women presenting for delivery at Hospital Universitario Japones and Centro de Salud 18 de Marzo in Santa Cruz for blood sample collection from the mothers and blood and urine sample collection from their newborns. Consent was obtained by trained study nurses. All survey, observation, and laboratory analysis data were anonymized, numbered, coded, and kept strictly confidential. Nurses were blinded to the CD positivity or negativity of each infant; however, infants confirmed to be positive for CD were referred to another physician for treatment.

### 2.2. Study design and human samples

Samples were collected between 2018 and 2020. After obtaining written informed consent, nurses collected blood from pregnant women presenting for delivery at Hospital Universitario Japones and Centro de Salud 18 de Marzo in Santa Cruz, where CD is endemic; this blood was screened for CD via the indirect hemagglutination test (PolyChaco, sensitivity and specificity according to manufacturer’s instructions were 98% and 99%, respectively), Trypanosoma Detect, an immunochromatographic strip assay (InBios International) (Sensitivity: 90.7%, Specificity: 100%), and via Chagatest ELISA with *T. cruzi* cytoplasmic and membrane antigens (Wiener Laboratories, Argentina. Sensitivity: 100%, and Specificity: 99.6%). If at least one of these tests returned a positive result, the mother was considered positive for CD. Blood was collected from each child born to this group of CD-positive women at 0, 1, 6, and 9 months after birth and subjected to testing.

The samples collected at 0 and 1 months underwent both qPCR and micromethod, in which the blood is centrifuged and the buffy coat layer is microscopically examined for the presence of *T. cruzi*. Samples collected at 6 and 9 months were subjected to at least two of the following serology tests: Chagatest Recombinante ELISA (Wiener Laboratories, Argentina. Sensitivity: 99.3%, and Specificity: 100%), Chagatest ELISA with *T. cruzi* cytoplasmic and membrane antigens (Wiener Laboratories, Argentina. Sensitivity: 100%, and Specificity: 99.6%), and the IHA (PolyChaco). Infants were considered CD-positive if microscopy or qPCR done on 0- or 1-month specimens gave positive results or if at least two serological tests done on 6- or 9-month samples were positive, and CD-positive infants were referred to physicians of the Bolivian National Chagas Disease Control Program for treatment.

Urine samples were also collected from all infants 1 month and 9 months after birth. These urine samples were frozen and sent first to Johns Hopkins University, then to George Mason University and stored at -80°C. The samples included in this study are 27 urine samples collected from CD-negative infants and 29 urine samples collected from congenitally infected, CD-positive infants (see Table 1). All infants included in the study had mothers who were considered CD-positive.

**Table 1:** Clinical characteristics of the samples utilized in the present study. Donating infants who tested positive for at least one CD test at birth considered positive congenital infection samples. All infants included in the study had CD-positive mothers. Comparison between positive and negative sample groups reveals broad similarities in terms of clinical details available. Mothers who reported their age ranged from 17-39 years of age.

	Positive samples (n = 27)	Negative samples (n = 29)	Total (n = 56)	P-value
Infant positive for at least 1 test at birth	27 (100%)	0 (0%)	27 (48%)	<0.0001 (Unpaired t test, two-tailed)
Infant positive for at least 1 test at 1 month	13 (48%)	0 (0%)	13 (23.2%)	<0.0001 (Unpaired t test, two-tailed)
Infant not tested at 1 month	2 (7.4%)	3 (10.3%)	5 (8.9%)	>0.9999 (Fisher’s exact test, two-sided)
Mother positive for 1 test	2 (7.4%)	2 (6.9%)	4 (7.1%)	>0.9999 (Fisher’s exact test, two-sided)
Mother positive for 2 tests	18 (66.6%)	20 (69%)	38 (67.8%)	>0.9999 (Fisher’s exact test, two-sided)
Mother positive for 3 tests	7 (25.9%)	7 (24.1%)	14 (25%)	>0.9999 (Fisher’s exact test, two-sided)

Urine was collected under informed consent from five CD-negative individuals in the United States to perform methodological development and to provide a geographically diverse set of negative controls.

### 2.3. Preparation of affinity capture materials

Affinity capture nanoparticles were prepared as previously described by Tamburro et al. Briefly, hydrogel nanoparticles composed of poly(N-isopropylacrylamide-co-acrylic acid) were synthesized by precipitation polymerization [27]. N-isopropylacrylamide (NIPAm) and N,N-methylenebisacrylamide were dissolved in water, filtered, purged with nitrogen, then combined with acrylic acid. Polymerization was induced by the addition of potassium persulfate and allowed to proceed overnight. The resulting particles were repeatedly washed in water via centrifugation to remove residual NIPAm [28].

Particles were then functionalized with the textile dye Remazol Brilliant Blue (RBB) via amidation chemistry as described by Tamburro et al. [27]. Briefly, this procedure was carried out by dissolving the dye in aqueous sodium carbonate, then combining the dye solution with the nanoparticles and stirring the reaction at room temperature for 48 hours. The resulting functionalized particles were washed in water via centrifugation to remove residual unbound dye [28].

The nylon affinity net was prepared as previously described by Cornero et al. [28]. Briefly, Nylon 66 mesh sheets were obtained from Pellon, Clearwater, FL. 0.5 g of nylon was pre-cleaned by immersing it in 50 mL of a 1% (w/v) Alconox detergent solution for 30 minutes with gentle agitation. After cleaning, residual detergent was removed by rinsing the nylon mesh five times with 50 mL of fresh deionized water per rinse. The cleaned mesh were then dried at 37°C overnight. The dye bath was prepared by dissolving 0.15 g of Sudan IV and 0.02 g of Alconox, in 15 mL of deionized water. The pH of the solution was adjusted to 4.5 using glacial acetic acid. The cleaned and dried nylon mesh was immersed in the dye bath, which was heated from 25°C to 100°C in 1 hour, maintained at 100°C for 1 hour, and cooled to 25°C in a TD130 IR Lab Dyeing Machine (ATI Corporation, New Holland, PA). Excess dye was removed by rinsing the affinity mesh twice with 50 mL of 0.1% (w/v) Alconox solution, followed by five rinses with 50 mL of deionized water, or until the rinse water ran clear. The dyed sheets were air-dried at 37°C overnight and stored for further use [29].

### 2.4. Mass spectrometry analysis

**Fig 1:**
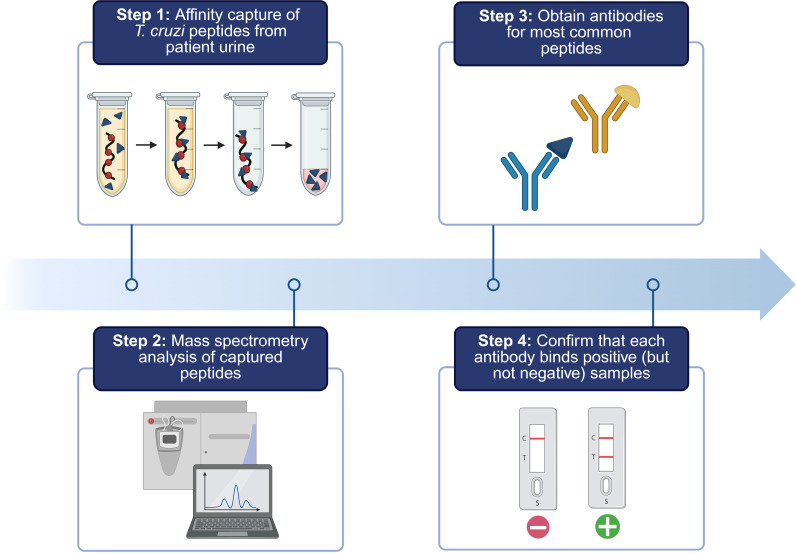
Overview of workflow. Identification of peptide fragments in patient urine via discovery mass spectrometry is used to prioritize *T. cruzi* proteins that may serve as useful biomarkers for new diagnostics. Antibodies raised against the highest priority candidates are revalidated in patient urine, test characteristics are reported, and proof-of-concept transition to point-of-care test is shown. Created in BioRender. Cassels, K. (2025) https://BioRender.com/p61q360.

14 CD-positive and 14 CD-negative samples were concentrated with the RBB nanoparticles and subjected to mass spectrometry analysis, as shown in Fig 1 (S1 Fig for a detailed diagram of which samples were used for each portion of the study). A calibrator was built by spiking *T. cruzi* strain CL Brener lysate (BEI resources) into urine of a healthy CD-negative adult. 100 μL of RBB particles suspension (7 mg/mL dry weigh concentration) were washed with ultrapure water to remove any free dye then incubated with CD-positive urine samples and calibrators (1 mL) for 30 minutes under rotation. The samples were then centrifuged (16,000 x g, 10 minutes, 25°C) to pellet the particles and supernatant was removed. Pellets were washed in 1 mL ultrapure water via centrifugation (16,000 x g, 5 minutes, 25°C). The particles were then lysed to elute concentrated peptides using 30 μL of 4% sodium dodecyl sulfate diluted in 50 mM ammonium bicarbonate and heated at 99°C for 10 minutes, yielding a concentrated solution of peptide analytes.

To prepare concentrated peptide solutions for analysis via mass spectrometry, the detergent was removed using ThermoFisher Pierce Detergent Removal Columns according to manufacturer instructions. Briefly, storage buffer was removed from the columns, and the columns were equilibrated with the addition of 0.4 mL phosphate-buffered saline (PBS, Fisher), followed by addition of sample to the column and centrifugation (1,500 x g, 2 minutes, 25°C). Eluates were saved and treated with urea to a concentration of 2 M to denature proteins, dithiothreitol to a concentration of 10 mM to reduce disulfide bonds, and iodoacetamide to a concentration of 50 mM to alkylate cysteine residues. Ammonium bicarbonate was then added to a concentration of 50 mM and trypsin was added in a 1:10 protease: protein ratio, then samples were incubated at 37°C overnight. The next day, 2 μL of undiluted trifluoroacetic acid were added to stop the digestion; samples were desalted using C18 columns (Pierce) according to manufacturer instructions. After elution from the C18 column, samples were dried under nitrogen flow and stored at -80°C until mass spectrometry analysis.

Dried, concentrated samples were reconstituted in 0.1% formic acid before 2 μL were injected into an Orbitrap Fusion Tribrid Mass Spectrometer coupled with a nanospray EASY-nLC 1200 UHPLC. Reversed-phase chromatography separation of the peptide mixture was performed using PepMap RSLC 75 μm i.d. × 15 cm long with 2 μm, C18 resin LC column with 0.1% formic acid for mobile phase A and 0.1% formic acid, 80% acetonitrile for mobile phase B. Peptides were eluted using a linear gradient of 5% mobile phase B to 50% mobile phase B in 90 minutes at 300 nL/min, then to 100% mobile phase B for an additional 2 minutes. The mass spectrometer was operated in a data-dependent mode in which each full MS scan was followed by TopN MS/MS scans of the most abundant molecular ions with charge states form 2+ to 4+ , which were dynamically selected for collision induced dissociation using a normalized collision energy of 35%.

Mass spectrometry parameters were chosen based on work validated by Magni et al. in their evaluation of urinary biomarkers for tick-borne illnesses [24]. The peptide false discovery rate based on searches of the decoy database was 1–5%. The precursor ion mass tolerance was < 5 ppm. The fragment mass tolerance was < 0.5 Da. The Q-value and delta Cn were less than <0.05 [24].

To interpret the results of this analysis, discovered peptides were searched against the Uniprot *T. cruzi* database in Proteome Discoverer (version 2.4), with the human and *E. coli* databases designated as decoy databases. To be considered, a peptide had to be greater than 7 amino acids in length and be a 100% match to *T. cruzi* and less than a 100% match to other organisms.

### 2.5. Verification of urinary peptides using Parallel Reaction Monitoring

Urine samples from 4 CD-positive infants were processed for mass spectrometry Parallel Reaction Monitoring (PRM, see S1 Fig). Peptide sequences were selected for compatibility with PRM according to established guidelines [30]. Samples were processed using the RBB nanoparticles and trypsin digestion as described above [31]. Briefly, nitrogen-dried samples, obtained as described in the Methods 2.4 section, were reconstituted in 0.1% formic acid and each patient sample was injected into an Orbitrap Exploris 480 Mass Spectrometer equipped with a nanospray 266 EASY-nLC 1200 HPLC system (ThermoFisher). Peptides were separated using a reversed-phase PepMap RSLC 75 μm i.d. × 15 cm long with 2 μm, C18 resin LC column (ThermoFisher). The mobile phases consisted of 0.1% aqueous formic acid (mobile phase A) and 0.1% formic acid in 80% acetonitrile (mobile phase B). After sample injection, the peptides were eluted by using a linear gradient from 5% to 50% B over 15 minutes and ramping to 100% B for an additional 2 minutes. The flow rate was set at 300 nL/min. The Orbitrap Exploris was operated using parallel reaction monitoring, in which only the precursor m/z values of the peptides of interest were permitted to pass through to be fragmented by HCD. Fragment ions were detected in the Orbitrap with resolution at 60,000. Data were analyzed with Skyline v3.6 (University of Washington, MacCoss Lab) to determine the presence or absence of peptides of interest.

### 2.6. Selection of candidate biomarkers and generation of monoclonal antibodies

The *T. cruzi* protein families detected in patient urine were recorded, and two of the most commonly appearing families were identified as trans-sialidase (TS) and mucin associated surface proteins (MASP). The conserved domains of each family were identified via literature and BLAST searches to design peptide antigens for the production of monoclonal antibodies. A designed consensus sequence was derived from the TS variable repeated region at the C-terminal end, consisting of the first two repeats of the 12-amino acid sequence that is repeated in this domain (residues 670–693 of Uniprot ID Q4CVS5, CL Brener strain). The final sequence CDSSAHGTPSTPVDSSAHGTPSTPV was used as the antigen for TS antibody generation. A designed consensus sequence derived from MASP (residues 281–310 of Uniprot ID Q4E2D3, CL Brener strain), with final sequence CTRTPDESDGSTAASHTTSPL, was used as the antigen for MASP antibody generation. Production of monoclonal antibodies targeting the conserved domains of these two proteins was contracted to Sino Biological. Briefly, Sino Biological synthesized and validated the antigens via SDS-PAGE and UV analysis, immunized mice (N = 5) with the antigens (3 rounds of immunization) and performed serum titer testing. The mouse with the highest titer value was selected for B-cell isolation and subsequent hybridoma generation. At least 5 hybridomas per antigen were generated, and purified antibodies from each clone were evaluated via ELISA and western blot. Clones with the highest sensitivity via dot blot were considered for further analysis. The antigen peptides against which the antibodies were raised were also provided by Sino Biological for antigen down immunoassay development, validation, and amplification to patient samples.

### 2.7. Antibody validation and limit of detection studies

Urine was collected from a CD-negative adult and clarified via centrifugation (3000 x g, 10 minutes, 25°C). Serial tenfold dilutions of both antigen peptides were then performed in the negative urine, and the dilutions were spotted in two 1 μL spots on polyvinylidene fluoride (PVDF) membrane pre-activated with methanol in a 10-fold dilution curve ranging from 100 ng antigen to 10 pg antigen. The resulting blots were blocked in 5% milk in PBS with 0.1% Tween (PBS-T) for 1 hour, then primary antibody was applied at a 1:1000 dilution in 5% milk in PBS-T overnight at 4°C. Blots were thoroughly washed in PBS-T, then secondary antibody (goat anti-mouse HRP conjugate, Jackson Immunoresearch 62-6520) was applied at a 1:10,000 dilution in 5% milk in PBS-T for 1 hour. Finally, SuperSignal™ West Dura Extended Duration Substrate (ThermoFisher) was added, and the membranes were imaged using chemiluminescent detection.

### 2.8. Detection of native antigen in *T. cruzi* lysate

The following reagents were obtained through BEI Resources, NIAID, NIH: Trypanosoma cruzi, Strain Brazil (+luc), NR-40347, and Trypanosoma cruzi, Strain CL, NR-49381. Strain Brazil (+luc) corresponds to discrete typing unit (DTU) I and was deposited as a trypomastigote; strain CL corresponds to DTU VI and was deposited as an epimastigote. Cells were harvested by centrifugation, washed with phosphate buffered saline, and lysed in 100 μL of RIPA buffer (ThermoFisher 89900) for 30 min on ice. Lysates were clarified via centrifugation and protein concentration was determined using Micro BCA Protein Assay Kit (ThermoFisher 23235).

The most sensitive antibodies against MASP and TS respectively were applied to a western blot containing *T. cruzi* strains Cl and Brazil, prepared as described above, which belong to the TcVI discrete typing unit (DTU) and TcI DTU respectively [32]. Lysate (5 μg) was loaded on a 4–20% tris-glycine gel (Life Technologies), which was then electrophoresed at 150 V for 1.5 hours. The proteins in the gel were transferred either at 25 V for 2 hours to a PVDF membrane pre-activated with methanol, or at 15V overnight at 4°C. The resulting blots were blocked and incubated with primary and secondary antibodies (dilution, diluting buffer), then incubated in SuperSignal™ West Dura Extended Duration Substrate (ThermoFisher) according to manufacturer instructions. Images were generated using chemiluminescent detection.

### 2.9. Antigen down immunoassay on patient samples

Nylon affinity mesh concentration and dot blot procedures were applied to 16 urine samples from CD-positive infants, 15 urine samples from CD-negative infants, and 4 urine samples from CD-negative adults (see S1 Fig), which were first thawed and clarified via centrifugation (3000 x g, 10 minutes, 25°C).

A total of 500 μL of each sample was incubated with a 10 mg nylon affinity net for 30 minutes, then samples were centrifuged (16,000 x g, 5 minutes, 25°C) and supernatant was decanted. The affinity net was washed in 500 μL water, then captured peptides were eluted with 40 μL elution buffer (3 M urea, 4.86 mM n-Octyl-β-D-thioglucopyranoside). The resulting eluates were then spotted onto dot blots using the procedure described above. Images were generated using chemiluminescent detection.

Each of these samples was spotted in duplicate in three 1 μL applications per spot onto PVDF membrane pre-activated with methanol. The resulting blots were blocked in 5% milk in PBS-T for 1 hour, then the corresponding primary antibody was applied at a 1:500 dilution in 5% milk in PBS-T overnight at 4°C. Blots were thoroughly washed in PBS-T, then goat anti-mouse secondary antibody was applied at a 1:10,000 dilution in 5% milk in PBS-T for 1 hour. Blots were then washed in PBS-T and incubated in SuperSignal™ West Dura Extended Duration Substrate (ThermoFisher) according to manufacturer instructions. Images were generated using chemiluminescent detection.

### 2.10. Lateral flow immunoassay

Lateral flow immunoassay (LFI) was developed as a competitive test in which labelled antigen competes with native antigen in the patient sample for gold-nanoparticle (NP)-labelled SBMASP4 antibody. The assay was developed using the Universal Lateral Flow Assay kit (Abcam ab270537) with some modifications to protocols. Briefly, a 0.1 mg/mL solution of SBMASP4 antibody was prepared in the included antibody diluent, and 1.2 μg was conjugated to gold-NPs following the manufacturer’s directions. After conjugation, antibody-labelled gold-NPs were separated from unlabeled antibody via centrifugation at 9000 x g for 10 min, washed twice with 10% quench buffer (as provided in ab270537), collected via centrifugation, and resuspended in 10% quench buffer to an optical density (OD) of 20. To label the MASP antigen, 10 μg of antigen was processed using the Biotinylation Kit/ Biotin Conjugation Kit (Fast, Type A) - Lightning-Link (Abcam ab201795) according to the manufacturer’s directions. Prior to running the LFI, both gold-NP labelled antibody and biotin-labelled antigen were diluted to OD 4 and 1 ng/uL working stocks, respectively, in 1X Universal Assay Running Buffer (as provided in ab270537) supplemented with 0.1% BSA. For optimization studies, samples were prepared by mixing 80 μL of test solution with 5 μL of labeled antibody, 5 μL of labeled antigen and incubating this solution at RT for 15 min. From this sample, 80 uL was added to a microtiter plate and the LFI strip (as provided in ab201795) was inserted and allowed to wick for at least 20 min prior to imaging of the strip. For urine dilution studies, urine from a CD-negative adult was diluted in PBS. For patient samples and competition studies, 5 CD-positive and 4 CD-negative samples were used (see S1 Fig). 80 μL of diluted patient urine or 80 μL of diluted negative urine spiked with unlabeled antigen was mixed 5 μL of labelled antibody and allowed to incubate for 5 min. Then, 5 μL of labelled antigen was added, and samples were further incubated for 10 min prior to application to the LFI strip.

### 2.11. Statistical analyses

Known CD-positive samples (N = 16) and known CD-negative samples (N = 19) were analyzed via dot blot analysis as described. Spot intensities were quantified using ImageJ, where each patient was analyzed on two independent blots, with each patient spotted in duplicate on each blot. Spot intensities per patient were averaged and utilized to determine receive operator characteristic (ROC) curves using GraphPad Prism version 10.2.1 with 95% confidence intervals calculated via the method Wilson/Brown for each antibody. Intensity cut-off values were chosen for each antibody to best balance sensitivity and specificity.

## 3. Results

### 3.1. Clinical and demographic patient information

Samples were collected from infants born in Santa Cruz, Bolivia to CD-positive mothers, who each received a positive result from at least one diagnostic test for CD. Infant blood samples were tested by a combination of qPCR, micromethod, Chagatest Recombinante ELISA, Chagatest ELISA with *T. cruzi* cytoplasmic and membrane antigens, and indirect hemagglutination test. Mothers underwent blood screening for CD via the indirect hemagglutination test, Trypanosoma Detect (an immunochromatographic strip assay), and Chagatest ELISA. Mothers ranged in age from 17 to 40 (see Table 1). It is estimated that at least 25% of women are positive for CD in Bolivia. The congenital transmission rate is estimated to be 6% [33].

### 3.2. *T. cruzi* proteins identified in infant samples via discovery mass spectrometry

For peptide discovery mass spectrometry, 14 CD-positive and 14 CD-negative samples were used (S1 Fig).

**Fig 2:**
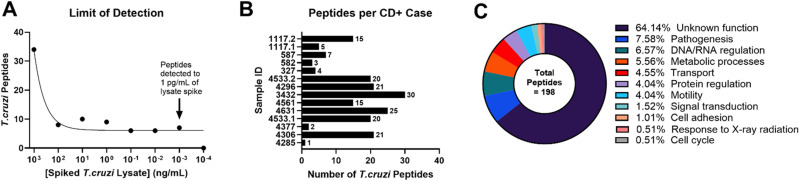
Identification of *T. cruzi* peptides in the urine of congenitally infected infants with Chagas disease. **A)** Serial ten-fold dilutions of *T. cruzi* lysate were prepared in CD-negative donor urine and analyzed using the protein mass spectrometry protocol *T. cruzi* peptides were detected in as little as 1 pg/mL of spiked lysate. **B)** Unique peptides identified in 14 CD+ congenitally infected infants which map to *T. cruzi.* Patients show a wide range in peptide counts. **C)** *T. cruzi* proteins for which a derived peptide was identified in patient urine were categorized according to molecular function. The vast majority of identified peptides were derived from proteins of unknown function, which could be due to poor genome annotation of *T. cruzi*. The second largest category of proteins identified were those related to parasite pathogenesis.

The method and instrument’s limit of detection was assessed to determine the likelihood of detecting all peptides present in the urine samples. *T. cruzi* strain CL Brener lysate (Biodefense and Emerging Infections Research Resources Repository), which belongs to the TcVI DTU, was spiked in healthy individuals’ urine at different concentrations (range 1000 ng/mL to 0.1 pg/mL) and was subjected to sample preparation as described above. *T. cruzi*-derived proteins were detected in the complex matrix of urine in a concentration as low as 1 pg/mL (see Fig 2A).

**Fig 3:**
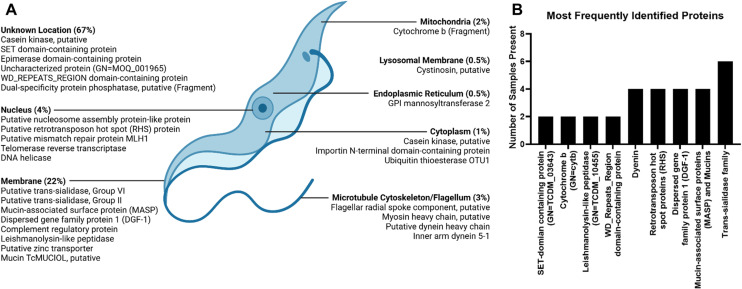
List of proteins identified in patient urine samples categorized by location in the parasite or by frequency of identification. **A)** The proteins listed were all identified via protein mass spectrometry in patient urine samples, and their locations in the parasite were determined by BLAST and literature search. Most of the identified proteins belong to an unknown location in *T. cruzi*. **B)** Most frequently identified proteins or family of proteins in CD+ congenitally infected infants map to the largest gene families in *T. cruzi*. Created in BioRender. Still, A. (2025) https://BioRender.com/f85d580.

Urine samples from 14 CD-positive and 13 CD-negative infants born to CD-positive mothers in endemic areas, along with 1 urine sample from a CD-negative adult, were analyzed using nanoparticle-based affinity capture methods to concentrate any antigens present. A total of 198 *T. cruzi*-derived peptides were identified in the 14 CD-positive patient samples with an average of 13 peptides per sample (see Fig 2B). Any peptides identified in CD-negative patient samples were included in the contaminant database. The majority of proteins are uncharacterized with unknown function; the most represented functional group is pathogenesis (see Fig 2C). Of those proteins with known function and location, most are localized on the membrane (22%) followed by the nucleus (4%). The following protein groups were highly represented in the analyzed urinary peptidome: trans-sialidases (TS), mucin associated surface proteins (MASP), dispersed gene family 1 (DGF-1), and retrotransposon hot spot proteins (see Fig 3). Peptides from those groups were chosen for verification through orthogonal methods.

For a full list of peptides identified in each sample, along with probable parent proteins, please see Table A in S1 Data.

### 3.3. Parallel reaction monitoring (PRM) verification of peptides identified in discovery phase

Discovery mass spectrometry data were verified using parallel reaction monitoring (PRM). PRM is a mass spectrometry technique used to detect the presence of specific peptides with high sensitivity and selectivity by monitoring all fragment ions of a precursor ion. It is considered orthogonal to discovery acquisition mode, under which the instrument scans a wider mass range to identify as many peptides as possible in a sample, without preselecting specific targets. Verifying discovery data enhances their generalizability to larger patient cohorts by reducing technological bias.

**Fig 4:**
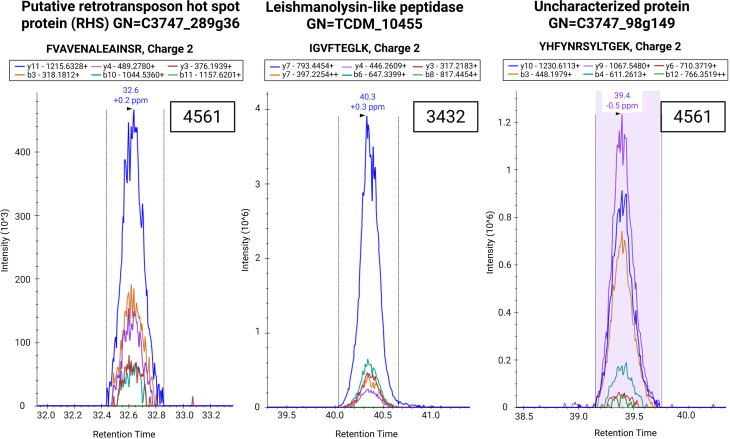
Verification of three peptides by PRM. Previously identified *T. cruzi* peptides from discovery mass spectrometry are validated via parallel reaction monitoring in a separate cohort of patient samples. Created in BioRender. Cassels, K. (2025) https://BioRender.com/1wqwtx7.

Parallel Reaction Monitoring (PRM) was used to verify the presence of 3 peptides, obtained in discovery mass spectrometry, in the urine of 4 CD-positive infant samples (see Fig 4). A filter of ≤ 1 ppm mass accuracy was applied to the product ions to allow for high-confidence peptide identification. Peptide FVAVENALEAINSR (retrotransposon hot spot protein) was detected in one sample and was representative of the retrotransposon hot spot protein family. Peptide IGVFTEGLK, belonging to the protein leishmanolysin-like peptidase, was found in two patient samples in the discovery phase; this protein sequence classifies it as similar to a GP63 metalloprotease family member from *Leishmania* species. GP63 protease family members are recognized virulence factors in *Leishmania* responsible for host cell attachment; they are thought to serve a similar function in *Trypanosoma*, although they may also regulate interactions with the insect vector as well [34,35]. Finally, the peptide YHFYNRSYLTGEK was identified in four patient samples. This peptide belongs to a *T. cruzi* protein for which a function has not yet been identified, although it does seem abundant in the urine of CD-positive patients. Overall, the peptides identified in this study may represent additional protein biomarker candidates present in urine beyond the most abundant gene products and provide additional candidates for future diagnostic efforts. The verification of these peptides in patient samples using a technique orthogonal to discovery mass spectrometry strengthens their generalizability and minimizes the risk of technological bias. Peptides successfully validated via PRM are highlighted in yellow in Table A in S1 Data.

### 3.4. Prioritization of biomarkers for downstream diagnostic development

**Fig 5:**
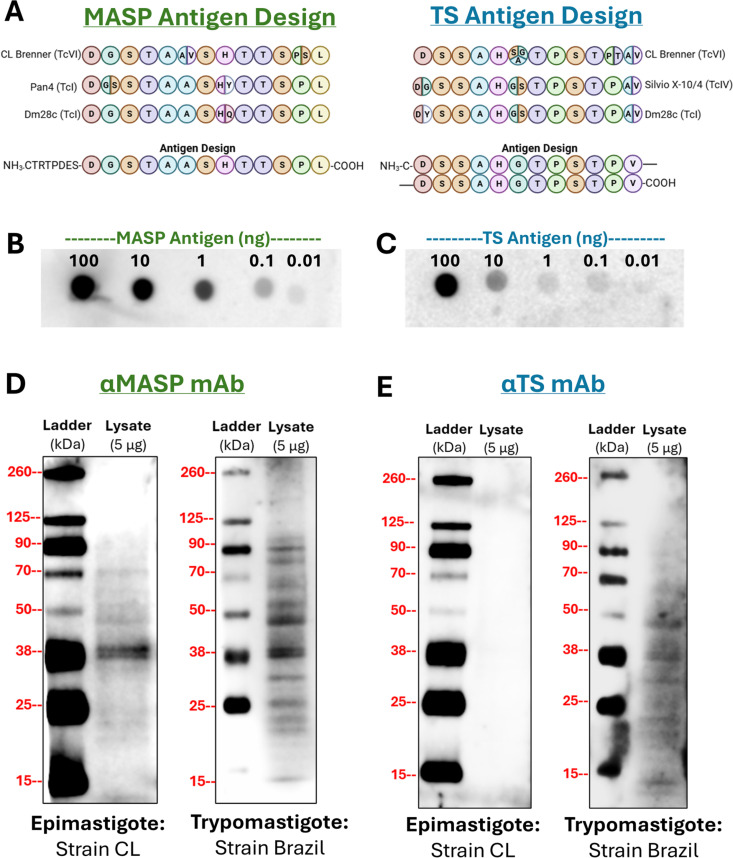
Design and validation of mAbs raised against conserved sequences of MASP and trans-sialidase protein families. **A)** Design of MASP (left) and TS (right) peptide antigens based on broadly conserved regions of each protein family. MASP antigen is derived from conserved C-term region, and TS antigen is derived from the immunogenic SAPA repeat region. Created in BioRender. Still, A. (2025) https://BioRender.com/y98s948 **B)** Best-performing αMASP mAb clone had sensitivity down to roughly 0.1 ng and performed with limited background in a dot blot. **C)** Best-performing αTS mAb clone had sensitivity down to roughly 1 ng and had slightly higher background in dot-blot. **D)** Probe of *T. cruzi* lysate in both the epimastigote (left) and trypomastigote (right) life cycles with αMASP mAb. **E)** Probe of *T. cruzi* lysate in both the epimastigote (left) and trypomastigote (right) life cycles with αTS mAb. SAPA repeats, against which the TS antibody is raised, are not found on TS family members in the epimastigote stage of the *T. cruzi* life cycle.

Two antigens were chosen as candidate biomarkers of CD positivity and proceeded to antibody production. These were selected from conserved domains of the TS and MASP protein families, which appeared frequently in patient urine samples according to mass spectrometry analysis (see Fig 3B). Antigen peptide sequences were chosen based on conservation within the protein family and across *T. cruzi* strains, as well as design concerns including hydrophobicity of the peptide and its expected antigenicity based on computational analysis and as reported in the literature. These antigens, as reported in Fig 5A, were prepared by Sino Biological for generation of multiple antibody clones against each. Antibody clones were screened via dot blot against the antigen to confirm their suitability for immunoassay. The respective antigen, MASP or TS, was prepared in a dilution curve in CD-negative donor urine to analyze limits of detections in a urine background. The best performing anti-MASP C-term mAb clone (SBMASP4) showed sensitive detection of antigen into the picogram range (Fig 5B), while the best performing antibody against TS SAPA has a sensitivity down to low nanograms (Fig 5C). To further confirm the potential utility of these antibodies for detection of native CD antigens in patient samples, both antibodies were used to probe *T. cruzi* lysates from both the epimastigote and trypomastigote life cycles of the parasite. Preliminary screening of the anti-MASP antibody against increasing quantities of *T. cruzi* CL lysate revealed a dose-dependent increase in both band intensity and detected bands, as expected (S2 Fig). As shown in Fig 5D, the anti-MASP antibody sensitively detected MASP family members in both epimastigote and trypomastigote life cycles, as expected, with increasing bands detected in the trypomastigote. This likely corresponds to the increasing diversity of molecular weights of MASP family members in this stage of the parasite life cycle, due to diverse sialylation patterns as well as increasing production of MASP family proteins [36–39]. In contrast, the SAPA repeat region of the trans-sialidase protein is not present on TS family members expressed in the epimastigote form, leading to an absence of TS bands in the epimastigote blot in Fig 5E [40,41]. However, a full ladder representing the many TS isoforms present in the trypomastigote life cycle of *T. cruzi* are visualized when blotting with strain Brazil. Full uncropped blots are available in S3 Fig. In conclusion, immunoassay studies demonstrated that our monoclonal antibodies, raised against conserved regions of MASP and TS, are strongly reactive against native *T. cruzi* proteins. This strong premise supports the use of these antibodies in patient samples.

### 3.5. Detection of antigens in patient samples

**Fig 6:**
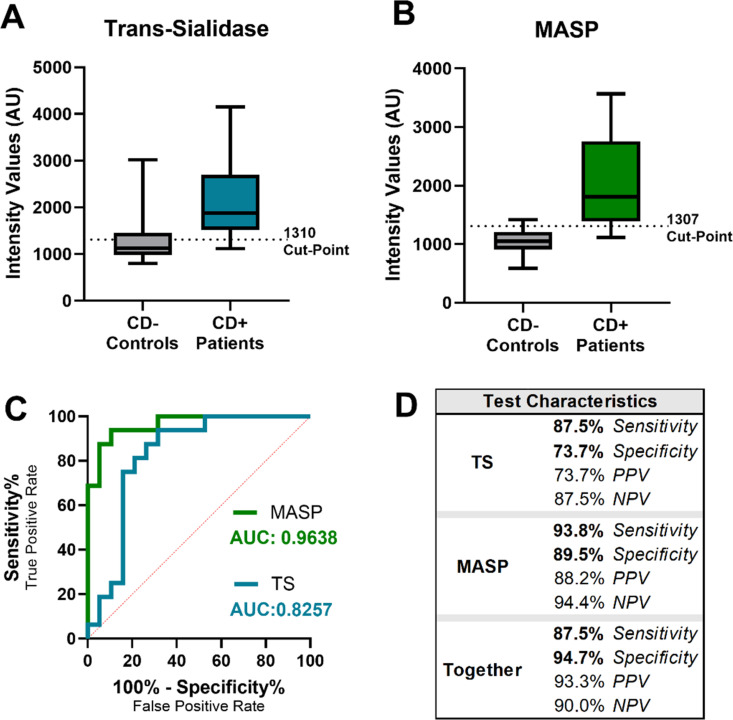
Discrimination between CD+ and CD- infant urine using newly generated anti-TS and anti-MASP antibodies. Data were derived from the blots in Fig 5. **A**) The trans sialidase antibody effectively differentiates positive and negative samples. **B)** The MASP antibody also discriminates between positive and negative samples. **C)** Receiver operating characteristic analysis shows an AUC of 0.8257 for the TS antibody and 0.9638 for the MASP antibody, indicating strong discrimination. **D)** Sensitivity and specificity for each antibody are reported, along with the performance of a combination test, which yields positive predictive values (PPV) and negative predictive values (NPV) of 90% or higher. In the combined test, a specimen is only considered positive if positive for both antibodies.

Following the validation of each antibody as sensitive for its antigen and suitable for use in a dot blot application, both antibodies were applied via immunoblot to 16 CD-positive urine samples and 19 CD-negative urine samples. Samples were concentrated using Sudan IV dye-functionalized nylon affinity net and eluted in 2M urea/0.3% n-Octyl β-d-thioglucopyranoside (OTG) based buffer compatible for spotting on the PVDF membrane [29]. Probing these nylon-concentrated patient eluates with both αMASP and αTS antibodies revealed increased spot intensities for positive patient samples over negative patient samples (S3 Fig). Intensity values for each sample were determined using ImageJ to calculate test performance as reported in Fig 6. A table showing the results by specimen for each antibody is available in Table B in S1 Data. The TS antibody demonstrated a sensitivity of 87.5% and a specificity of 73.7%, producing four false positive results and two false negative results. The MASP antibody demonstrated a sensitivity of 93.8% and a specificity of 89.5%, producing one false positive result and one false negative result. When both antibodies are considered together, and a sample is only considered to have a positive result if its intensity value falls above the positivity cutoff for both antibodies (i.e., a positive result is produced by both the anti-TS and anti-MASP antibody applications), the combined immunoassay has a sensitivity of 87.5% and a specificity of 94.7% (see Fig 6). Positive predictive value, or the likelihood of a positive result if a positive result exists, and negative predictive value, or the likelihood of a negative result if a negative result exists, are also included in Fig 6 as a more patient-centric measure of test efficacy.

### 3.6. Lateral flow immunoassay

**Fig 7:**
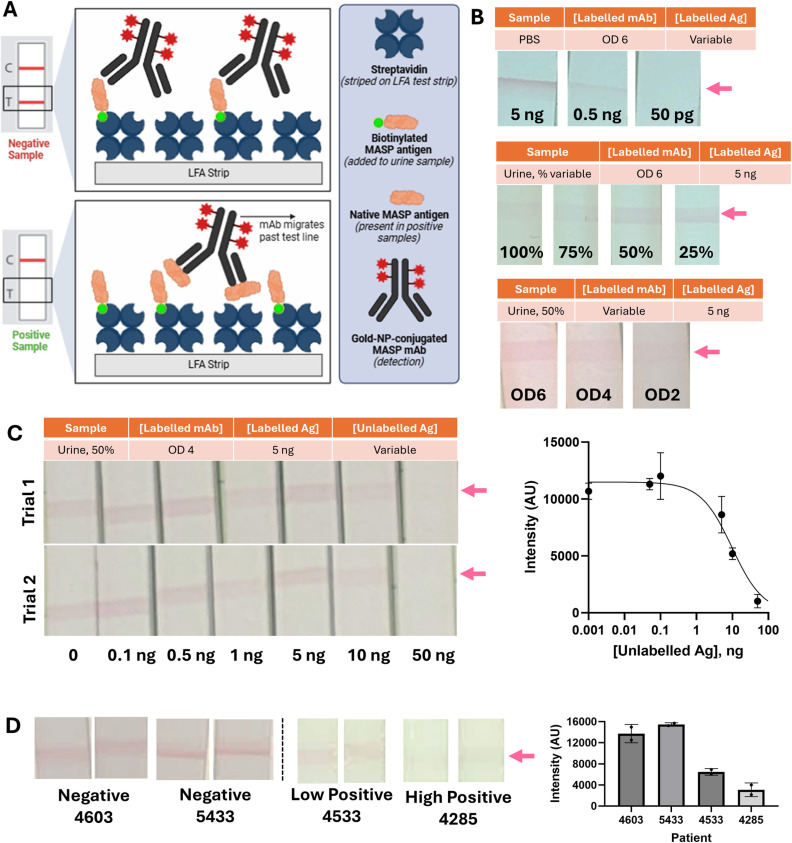
Proof-of concept competitive lateral flow immunoassay using αMASP mAb shows complete reduction in band intensity in urine solutions of roughly 1 ng/μL. **A)** Design of proof-of-concept lateral flow immunoassay; reductions in test line band intensity are expected in the presence of antigen in the test sample. Created in BioRender. Still, A. (2025) https://BioRender.com/x82y575 **B)** Optimization of lateral flow design shows that the test performs well in negative urine utilizing 5 ng of labelled MASP Ag, dilution of test urine to 50% in PBS to reduce background, and labelled mAb-Gold NP diluted to an optical density of 4. Test utilizes 40 μL of urine. **C)** Using optimized test conditions, increasing quantities of unlabeled MASP Ag were spiked into negative control urine. A reduction in band intensity of 50% is observed with 10 ng of spiked antigen; complete band reduction is achieved by 50 ng. Complete band reduction is therefore achieved a roughly 1 ng/μL starting concentrations of MASP antigen in urine. Quantitation of band densities via ImageJ shown in dose-response plot at right; 0 ng antigen quantitation displayed as 0.001 ng and data fit to a sigmoidal dose curve for visualization. **D)** Negative patients show the expected test lines (left), while positive patients can show reductions in test line band intensity (right). Inter-patient variability in test line intensity (compare negatives 4603 and 5433), due to urine matrix effects, must be overcome prior to larger screening of patient cohorts. Quantification of band densities via ImageJ shown at right.

The test promising early performance (Fig 6) and the need for a diagnostic modality deployable to the point of care prompted us to further evaluate the MASP antibody specifically for its ability to transition to a lateral flow immunoassay modality. This modality has advantages in resource-poor settings due to its ability to be utilized on-site, with same-day results, and without the need for highly trained laboratory personnel [42]. For a proof-of-concept evaluation, we developed a competitive lateral flow immunoassay in which labelled αMASP antibody produces a signal when binding labelled MASP antigen immobilized on the test line of the strip. Positive samples (S1 Fig), containing unlabeled MASP antigen, reduce signal intensity compared to negative samples (schematic provided in Fig 7A).

As shown in Fig 7, conjugation of gold-NPs to the anti-MASP antibody did not significantly reduce its ability to bind to the MASP antigen in phosphate buffered saline (PBS), with visual detection to 500 pg versus roughly 100 pg in direct dot blot, which includes washing steps and signal amplification via secondary antibody detection (Fig 7B versus Fig 5B). However, urinary background is non-trivial and does reduce sensitivity as compared to PBS; conducting the test in 50% diluted urine optimized sensitivity concerns with minimal concentration of labelled antibody and antigen (Fig 7B). Within this urinary background, unlabeled MASP antigen can successfully compete with labelled antigen to reduce test band intensity; complete elimination of test band is observed by 50 ng of unlabeled MASP antigen spiked into negative urine. With test volumes of 80 uL and 50% dilution of urine, this suggests the test can detect initial urinary MASP concentrations of approximately 1 ng/μL. Several patient urinary samples were tested with the proof-of-concept lateral flow immunoassay (Full images of lateral flow tests strips are provided in S4 Fig). Several CD-positive patient samples reduced band density as compared to negative samples, as shown in Fig 7D, suggesting that the lateral flow assay can be used in clinical samples. Analytical sensitivity of the assay could be achieved using affinity concentration of urine samples, as was conducted in Fig 6, which also reduces the urinary background signal [43], or via enhanced detection strategies [44]. In summary, our MASP monoclonal antibody was successfully incorporated in a lateral flow immunoassay that achieved dose-dependent response in a dynamic range relevant for human studies, and anecdotal evidence supports its ability to detect true positive cases using urine samples.

## 4. Discussion

Previously, three obstacles have hindered the development of diagnostic tests for CD in urine. First, biomarkers of the disease are low in abundance and diluted in a large volume of urine. Second, it has been difficult to identify antigens that will result in a high-specificity test. Finally, *T. cruzi* is so hypervariable that antibodies against a particular antigen may not react to every sample, given that that sample may contain the same protein with a slightly different sequence. In this paper, we have addressed each of these issues to present initial development of a diagnostic test based on two novel *T. cruzi* antigens, from the early stages of biomarker discovery to clinical proof of concept.

Using affinity-based concentration techniques, we were able to overcome the issue of dilution and create concentrated solutions of small analytes in high abundance. We were then able to use mass-spectrometry analysis to identify specific proteins present in the concentrated CD-positive patient samples and absent in concentrated CD-negative samples. Discovery mass spectrometry data were verified using parallel reaction monitoring (PRM). PRM is a mass spectrometry technique used to detect the presence of specific peptides with high sensitivity and selectivity by monitoring all fragment ions of a precursor ion [30]. In this data acquisition modality, the mass spectrometer isolates and fragments a target precursor ion, allowing for the simultaneous detection of all its fragment ions, thus capturing the entire fragmentation spectrum in a single scan. It is considered orthogonal to discovery acquisition mode, under which the instrument scans a wider mass range to identify as many peptides as possible in a sample, without preselecting specific targets. Verifying discovery data enhances their generalizability to larger patient cohorts by reducing the risk of technological bias. We most frequently identified members of the TS and MASP families in these samples. While the precise “most abundant” gene family in the *T. cruzi* parasite can vary in literature due to the complexity of the *Trypanosoma* genome, in general the five most abundant gene families are reported as the TS family, the MASP family, the retrotransposon hot spot family, the mucin family, and the DGF-1 family, in that order [37,40,45,46]. These also correspond to the most abundant protein families identified in our patient samples. In particular, the TS and MASP protein families appear in the largest number of positive samples and thus were identified as the highest priority potential urinary biomarkers of the presence of *T. cruzi*.

Trans-sialidases serve an immunomodulatory role and transfer sialic acid residues from the host cell surface proteins to parasite surface proteins, while the MASP family has an unknown function [36,47–52]. However, *T. cruzi* exhibits extreme genetic variability [36,49,50,53,54]. Thus, in order for a protein-based diagnostic test to detect all or the majority of strains of *T. cruzi* with which a patient may present, the peptide used to indicate CD-positivity must be well-conserved both within its own protein family as well as across strains of the parasite. While mass spectrometry provides a relatively unbiased look at proteins found in the patient urine, peptide characteristics required for MS detection (such as charge state or length) may not be present in a region of the protein most useful for antibody generation. Therefore, development of diagnostic assays using identified protein biomarkers as determined by mass spectrometry may need to target regions of the identified protein that are different from the precise sequence identified in mass spectrometry. The TS and MASP families are both highly variable but do possess conserved regions suitable for antibody generation, making them promising candidate biomarkers [54]. The two conserved regions of the TS family are the FLY domain, which is not well-conserved even within the TS family, and the Shed Acute Phase Antigen (SAPA) region [55,56]. The SAPA region consists of a variable number of repeats of a 12-amino acid sequence, is highly antigenic, well-conserved, and believed to be crucial to immune evasion [36,54,55,57,58]. As such, this peptide is likely to be present and detectable in CD-positive urine, regardless of the specific *T. cruzi* strain with which the patient is infected. The MASP conserved domain is the C-terminal region; this region is regularly cleaved from the rest of the protein and secreted by the parasite into the host bloodstream, making this peptide extremely likely to appear in positive patient urine in detectable quantities [37,56,59,60]. This region is also highly conserved across the MASP family and moderately well conserved across multiple *T. cruzi* strains [38,59]. Thus, these two sequences, as shown in Fig 5A, seem likely to be detectable across various CD-positive patient samples.

Given its antigenicity, SAPA has been used as the target of CD diagnostic assays by several other groups. Many studies have shown that the presence of SAPA in patient sera is a reliable indicator of CD positivity [61–65]. Of particular note is the work of Houghton et al., who successfully developed a lateral flow immunoassay for the detection of SAPA in serum samples; this test was intended for use in screening blood and organ donations [65]. Additionally, Castro-Sesquen et al. have validated the use of a diagnostic test relying on ELISA detection of anti-SAPA IgM in serum samples taken from newborns in endemic areas [62]. Our results align with these findings, and detection of CD by urinary antigens has previously been accomplished by our group and others [17,20,66,67]. However, to the best of our knowledge, this work is the first to identify SAPA specifically as a biomarker of congenital CD in human urine and utilize a monoclonal anti-SAPA antibody to differentiate congenital CD-positive and negative patients. The sensitivity of this test, which is often difficult to achieve in urinary diagnostics, is due in large part to expansion of the affinity concentration techniques discussed in our previous work [17]. Consistent, accurate detection of either the TS or MASP antigens in patient urine samples is not possible without the use of the nylon affinity net, as we demonstrate in the antigen-down immunoassay Fig 6 using concentration techniques and in the lateral flow immunoassay in Fig 7 without.

The MASP family, which is the second largest family expressed by *T. cruzi*, is also under study by many groups [51]. These are surface glycoproteins of unknown function, though they may be involved in mediating parasite-host cell interactions and are believed to help overwhelm and redirect the host immune response, similar to the function of TS [49–51,54]. These contain possible epitopes for B cells and MHC I and II, and this family has been identified as an important antigen [49,51,68]. Members of the MASP family seem to be cleaved into glycopeptides in the host bloodstream, some of which contain terminal sialic acid residues. This evidence suggests a path to the presence of the analytes in the urine [49,69,70]. The C-terminal domain of MASP has been identified in extracellular vesicles excreted by *T. cruzi*, and MASP antigens have been demonstrated to provoke an immune response in mice [38,68,71]. However, to our knowledge, no attempt has been made to use a MASP antigen as the basis for a CD diagnostic in any patient cohort, making the demonstration of a sensitive and specific MASP-based diagnostic a novel finding.

If newborn patient urine were tested via a direct test, ideally for multiple antigens given the complexity of the *T. cruzi* genome and the existence of many discrete typing units, the sensitivity and specificity of the test would likely improve. As shown by our results in Fig 6, the specificity of the test improves (increasing to 95%) when detection of both antigens is required for a positive result. Ideally, even more antigens would be included in such a panel of tests. Continued use of our biomarker discovery workflow could yield additional urinary *T. cruzi* antigens, which could form the basis for additional tests and improve the accuracy of CD detection. (In particular, the DGF-1 and retrotransposon hotspot proteins are already shown to be highly abundant in CD-positive urine.) The use of multiple antigens makes it more likely that even patients infected with different strains of *T. cruzi* (of which there are over 6000) would be correctly identified as CD-positive [72].

If such a panel of tests could be provided to every at-risk newborn, much greater numbers of congenitally infected newborns could be identified within the first year of life. Infants who receive treatment during that first year tolerate it extremely well and are mostly able to lead CD-free lives [33,62,73,74]. Efforts to provide treatment to infants that need it would be assisted by the accessibility of the above test. The use of this test in clinical applications could contribute greatly to the effort of identifying and treating congenital CD infections, which may account for one quarter of new CD cases each year and is the primary means of transmission in non-endemic countries, such as the United States [33,74–77].

While obstacles persist in the areas of patient access to care and treatment affordability and compliance, such an improvement in testing could go a long way in the reduction of the disease burden of CD. To further expand the utility of the MASP antigen-down immunoassay described in Fig 6, we describe proof of concept transition to a lateral flow immunoassay modality in Fig 7. While a sandwich immunoassay is preferred for clinical use due to more intuitive interpretation, this proof-of-concept let us determine the answers to a couple key questions. First, does the anti-MASP antibody retain sensitivity following conjugation to lateral flow detection elements such as gold nanoparticles? Second, can endogenous urinary MASP outcompete labelled MASP at reasonable concentrations, suggesting suitable affinity in the presence of urinary matrix? Thirdly and related, how significant are urinary matrix effects on test performance? Will a concentration step be strictly required for positive results, both to increase concentration of antigen but also to remove urinary background? We have found in many disease contexts that concentration of urinary antigens, as was done for samples processed in Fig 6, can be essential to test performance [24,78,79]. However, due to the high affinity of the MASP antibody produced (pg detection, as shown in Fig 5B), it was reasonable to determine whether this step was required. We find, in answer to the above questions, that the MASP antibody developed in this work both adapts well to the lateral flow immunoassay modality, with limited loss in sensitivity, and can detect antigen in patient samples, at least anecdotally. We do find that limited sensitivity in unconcentrated urine, and variable urinary background, strongly supports the use of a concentration step in further development of the lateral flow assay.

This study has several limitations. Given the difficulty of acquiring samples of congenitally infected infant urine, particularly the challenge of positively diagnosing CD given the lack of a gold-standard diagnostic, the sample sizes used in this study are relatively small. This small sample size places limits our ability to firmly determine the sensitivity and specificity of the discovered antibodies, and validation of these antibodies in a larger set of specimens would be required before the application of this assay as a clinically appropriate diagnostic. However, this limitation is partially ameliorated given the striking difference between signal in the positive and negative sample groups. Power calculations indicate that for the given average intensity values and standard deviation of each group, a sample size of 14 is sufficient to achieve discrimination between groups in the TS study with a p < 0.05 and a power of 80%. Therefore, group sizes of N = 16 positive samples and N = 19 negative samples do provide the necessarily statistical power to form the foundation of a CD diagnostic. Furthermore, the enhanced discrimination of the MASP antibody, mainly due to the reduced number of false positives as compared to the TS antibody, showed that with a power of 80% and p < 0.05, only 6 samples per group are required. A power of 95% can be achieved with group sizes of 10, criteria both our positive and negative samples groups meet. While our current findings are appropriate for antigen discovery, additional samples collected and used as confirmatory cohort will further strengthen the conclusions and refine test performance characteristics for both antibodies.

We present a proof of concept for a lateral flow assay based on our most promising antibody-antigen pair. The SBMASP4 antibody was successfully integrated into a lateral flow immunoassay, demonstrating a dose-dependent signal in urine within a dynamic range relevant to human studies. Additionally, it anecdotally detected true positive and true negative patients. Further verification and validation in larger patient cohorts are warranted.

To enhance assay sensitivity, future improvements will include coupling it with a sample concentration step, as successfully demonstrated by Paris et al. and incorporated in our antigen-down immunoassay presented in Fig 6 [79]. In this study, we introduce a novel sample concentration technology—the affinity nylon mesh—which offers significant advantages over previous methods. Unlike traditional approaches, it can be used at the point of need, incorporated into a urine collection device, and mechanically manipulated and separated with high efficiency, eliminating the need for centrifugation or magnetic separation. Due to its strong affinity for target molecules and superior separation efficiency, the affinity nylon mesh outperforms other concentration methods and holds promise as a bridge between field-based diagnostics and laboratory analysis.

## 5. Conclusion

This study utilized human samples to identify and verify *T. cruzi*-derived peptides in the urine of congenitally infected infants with CD. The molecular data guided the development of monoclonal antibodies that specifically reacted with the native *T. cruzi* antigen and were incorporated into a low-complexity laboratory immunoassay. This assay effectively distinguished CD-positive from CD-negative samples in a small cohort of infected infants. The antibodies were then incorporated into a lateral flow immunoassay, demonstrating a dose-dependent response down to 1 ng/μL in human urine and anecdotally differentiating CD-positive from CD-negative patients. These findings support the feasibility of developing widely deployable antigen-based diagnostics for congenital Chagas disease—an essential step toward improving timely detection of new cases and reducing the global burden of the disease.

## Supporting information

S1 Data Spreadsheet with individual peptides identified in discovery MS (Table A) and the per-specimen table with anti-MASP and anti-TS blot quantifications (Table B). (XLSX)

S1 Fig Diagram of CD-positive urine sample usage. A total of 27 CD-positive urine samples were used in this study; many were used for more than one phase of the study based on available sample volume. No negative samples were used more than once.
(TIF)

S2 Fig Dose-ranging studies and full uncropped blots from Fig 5. A) While MASP and TS are expected to visualize as a ladder at multiple molecular weights in western blot due the large protein family sizes, we confirmed the dose-dependency of this effect in preliminary dose-ranging studies. *T. cruzi* strain CL lysate (1 – 10 μg as indicated) was probed with 1:1000 dilution of anti-MASP antibody. As lysate quantity increases, additional MASP family member bands are visualized clearly. Multiple bands visualized are additional protein products from *T. cruzi* lysate, rather than background contaminating bands. **B)** *T. cruzi* strain CL blots shown in Fig 5 are shown uncropped. One gel was transferred to one membrane, which was cut in half and incubated in anti-TS and anti-MASP antibodies respectively, then imaged simultaneously to ensure equivalent run, transfer, and exposure conditions. Lanes that are cropped and shown in the main body of the paper are indicated with an arrow. **C)** *T. cruzi* strain Brazil blots shown in Fig 5 are shown uncropped. One gel was transferred to one membrane, which was cut into 2/3 and 1/3 and incubated in anti-TS and anti-MASP antibodies respectively, then imaged simultaneously to ensure equivalent run, transfer, and exposure conditions. Higher degrees of background are observed with anti-TS antibody.
(TIF)

S3 Fig Duplicate immunoblots showing antibodies against (A) TS and (B) MASP applied to patient samples that have been concentrated using nylon affinity net concentration techniques. The upper half of the blot contains positive samples, while the lower half of the blot contains negative samples (see S1 Data for a detailed sample list). Spot intensities were determined via ImageJ and used to determine the sensitivity and specificity of these antibodies reported in Fig 6.
(TIF)

S4 Fig Full images of lateral flow test strips. **A)** Spike-in study was conducted in negative patient urine (4603) in which increasing quantities of unlabeled MASP antigen are added to compete with labeled MASP antigen on the test line. Each concentration of unlabeled antigen was run in duplicate. Reduction in band intensity is observed with 10 ng of spike; test band is completely eliminated with 50 ng of spike. **B)** Four CD-negative infant urines were tested in duplicate with the above developed lateral flow immunoassay. All produced test bands; patient 4603 and 5433 are shown Fig 7. **C)** Five CD-positive infant urines were tested in duplicate using the lateral flow immunoassay. Patients 4533 and 4285 reduced band intensity and are shown in Fig 7.
(TIF)
