# Supplementary figures and images for: Mass spectrometry-based discovery and diagnostic validation of T. cruzi antigens in the urine of congenitally infected Chagas Disease patients
Source: PLoS Negl Trop Dis. 2025 Jun 16;19(6):e0013082. doi: 10.1371/journal.pntd.0013082 (PMC12169537; doi:10.1371/journal.pntd.0013082)

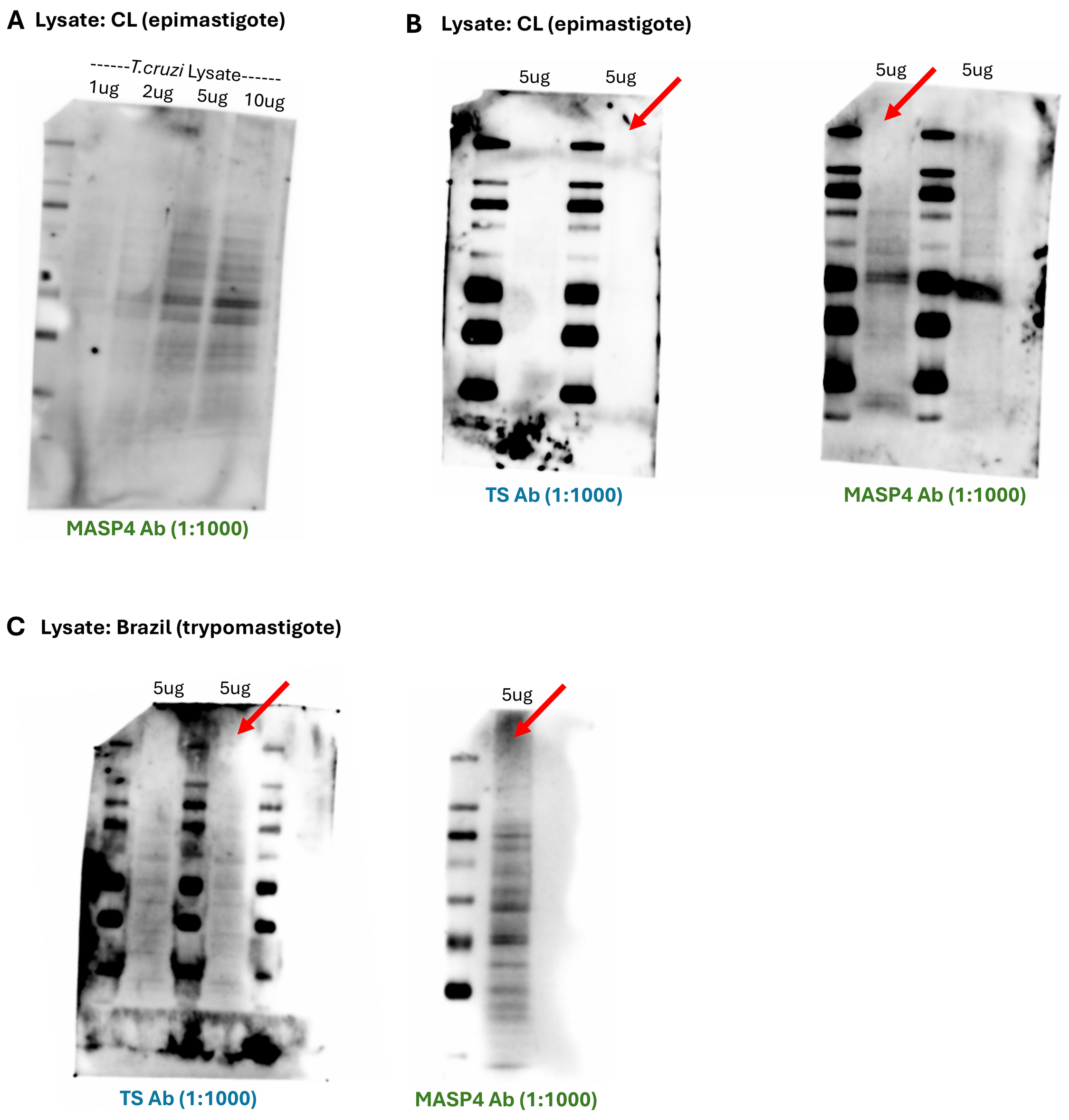

Supplement: S1 Fig — A total of 27 CD-positive urine samples were used in this study; many were used for more than one phase of the study based on available sample volume. No negative samples were used more than once. (TIF) [file pntd.0013082.s002.tif]

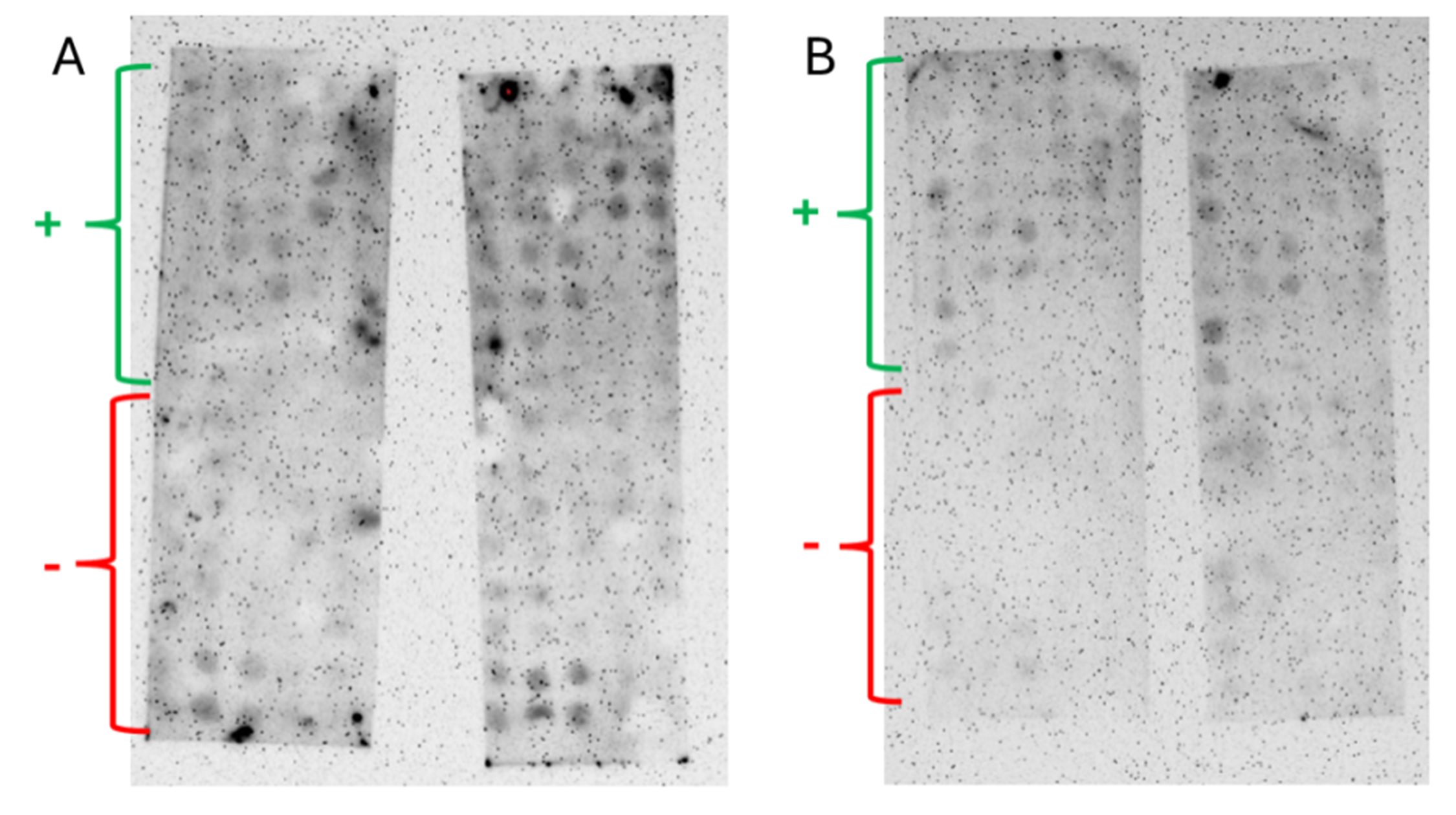

Supplement: S2 Fig — A) While MASP and TS are expected to visualize as a ladder at multiple molecular weights in western blot due the large protein family sizes, we confirmed the dose-dependency of this effect in preliminary dose-ranging studies. T. cruzi strain CL lysate (1 – 10 μg as indicated) was probed with 1:1000 dilution of anti-MASP antibody. As lysate quantity increases, additional MASP family member bands are visualized clearly. Multiple bands visualized are additional protein products from T. cruzi lysate, rather than background contaminating bands. B) T. cruzi strain CL blots shown in Fig 5 are shown uncropped. One gel was transferred to one membrane, which was cut in half and incubated in anti-TS and anti-MASP antibodies respectively, then imaged simultaneously to ensure equivalent run, transfer, and exposure conditions. Lanes that are cropped and shown in the main body of the paper are indicated with an arrow. C) T. cruzi strain Brazil blots shown in Fig 5 are shown uncropped. One gel was transferred to one membrane, which was cut into 2/3 and 1/3 and incubated in anti-TS and anti-MASP antibodies respectively, then imaged simultaneously to ensure equivalent run, transfer, and exposure conditions. Higher degrees of background are observed with anti-TS antibody. (TIF) [file pntd.0013082.s003.tif]

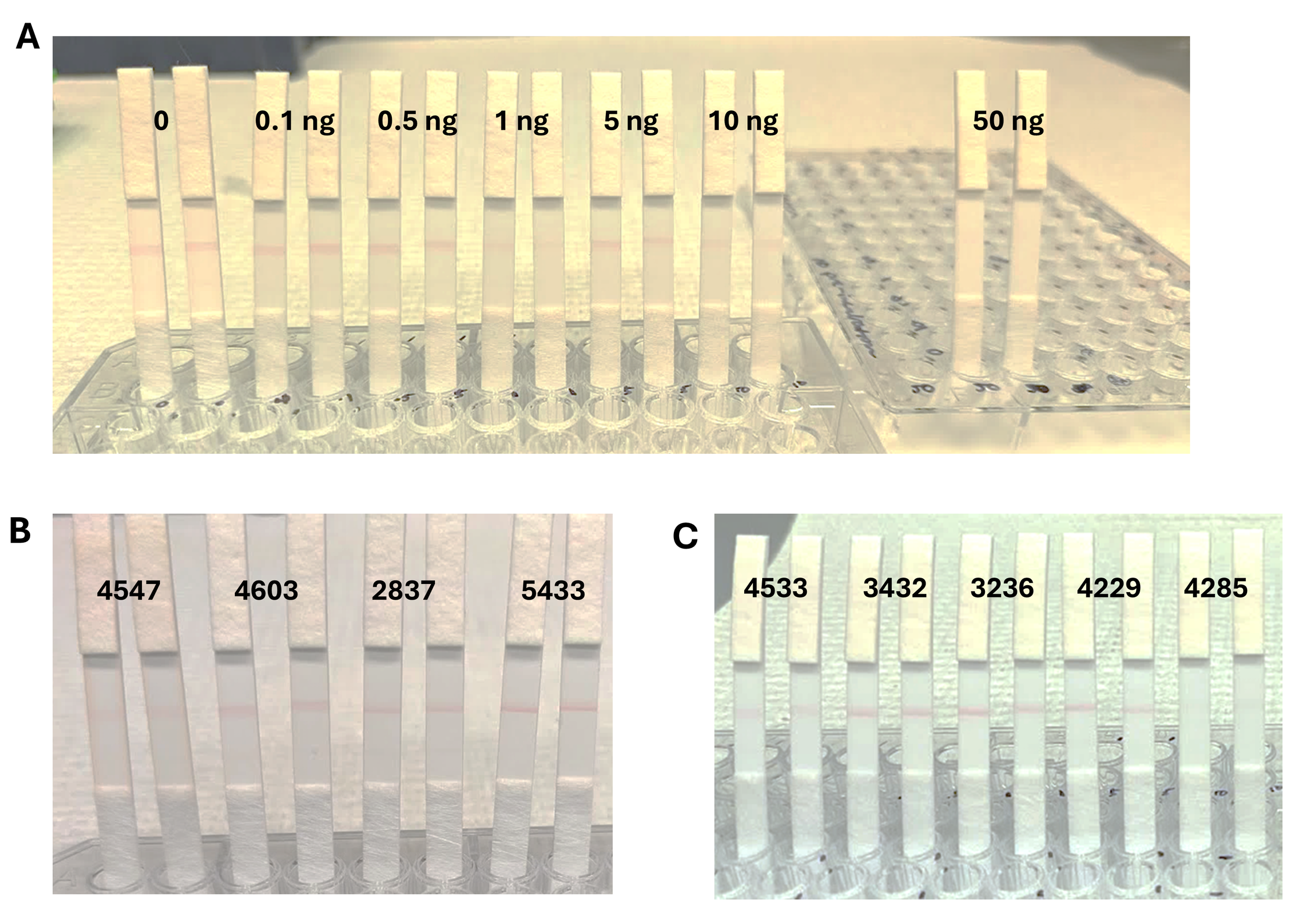

Supplement: S3 Fig — The upper half of the blot contains positive samples, while the lower half of the blot contains negative samples (see S1 Data for a detailed sample list). Spot intensities were determined via ImageJ and used to determine the sensitivity and specificity of these antibodies reported in Fig 6. (TIF) [file pntd.0013082.s004.tif]

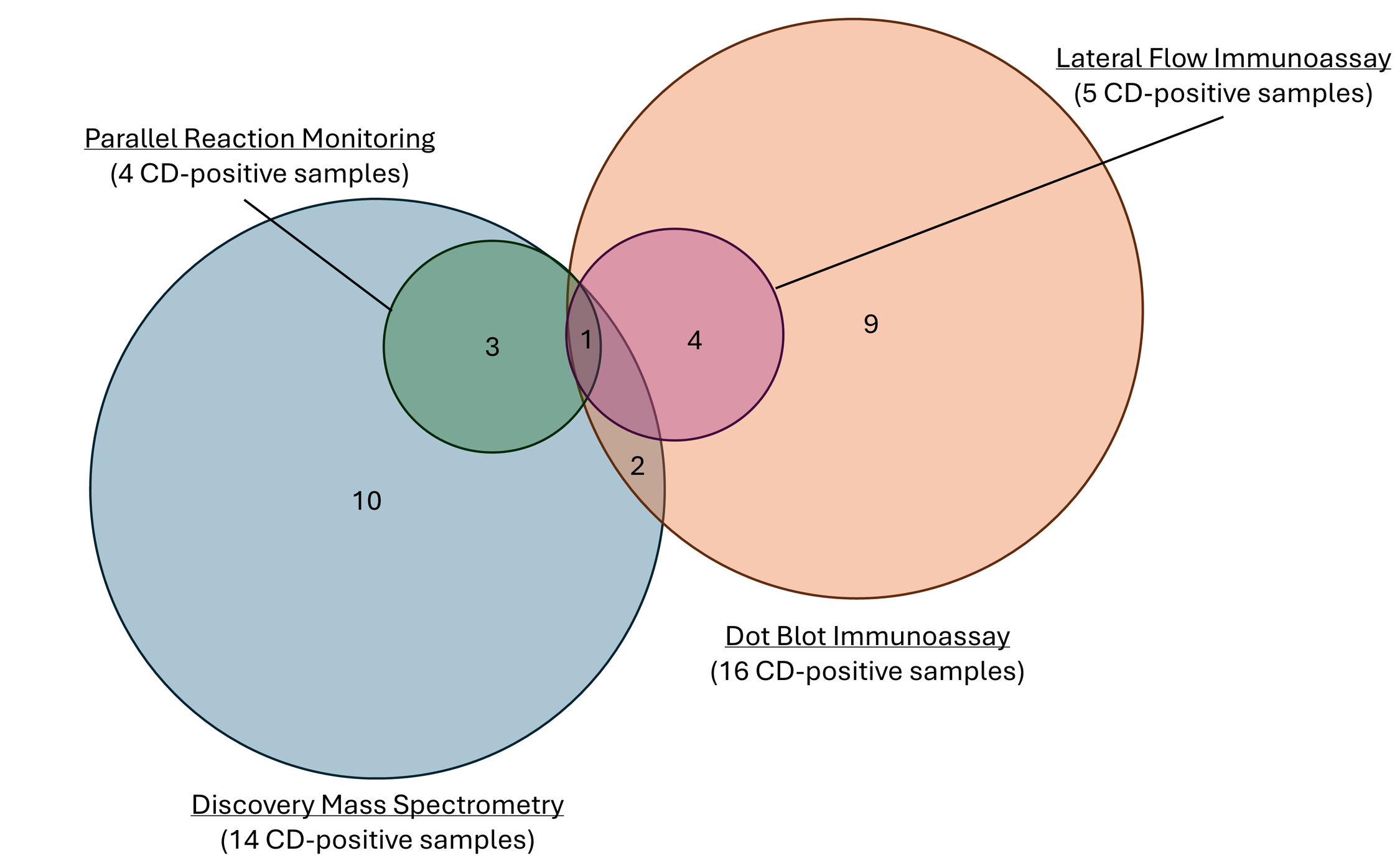

Supplement: S4 Fig — A) Spike-in study was conducted in negative patient urine (4603) in which increasing quantities of unlabeled MASP antigen are added to compete with labeled MASP antigen on the test line. Each concentration of unlabeled antigen was run in duplicate. Reduction in band intensity is observed with 10 ng of spike; test band is completely eliminated with 50 ng of spike. B) Four CD-negative infant urines were tested in duplicate with the above developed lateral flow immunoassay. All produced test bands; patient 4603 and 5433 are shown Fig 7. C) Five CD-positive infant urines were tested in duplicate using the lateral flow immunoassay. Patients 4533 and 4285 reduced band intensity and are shown in Fig 7. (TIF) [file pntd.0013082.s005.tif]
